# Tunable Multipolar Fano Resonances and Electric Field Enhancements in Au Ring-Disk Plasmonic Nanostructures

**DOI:** 10.3390/ma11091576

**Published:** 2018-09-01

**Authors:** Rong Qiu, Hang Lin, Jing Huang, Cuiping Liang, Zao Yi

**Affiliations:** 1 Joint Laboratory for Extreme Conditions Matter Properties, Southwest University of Science and Technology, Mianyang 621010, China; qiurong@swust.edu.cn (R.Q.); lh9711100@yeah.net (H.L.); h2311320325@yeah.net (J.H.); lcp144@yeah.net (C.L.); 2 Sichuan Civil-Military Integration Institute, SCII, Mianyang 621010, China

**Keywords:** Au ring-disk nanostructures, Fano resonances, multipolar, finite-difference time-domain

## Abstract

We theoretically research the characteristics of tunable multipolar Fano resonances in novel-designed Au ring-disk plasmonic nanostructures. We systematically study some structural parameters that influence the multipolar Fano resonances of the nanostructures. Adjustment of the radius (R_1_ and R_2_) of the Au ring, the radius (R_3_) of the Au disk and the thickness (H) of the Au ring-disk can effectively adjust the multipolar Fano resonances. The complex field distributions excited by a Au ring-disk can produce dark resonance modes. At the frequency of the multipolar Fano resonances, strong localized field distributions can be obtained. The Fano resonances exhibit strong light-extinction properties in Au ring-disk nanostructures, which can be applied to an optical tunable filter and optical switch.

## 1. Introduction

In recent years, because of their strong ability to control light and thereby inducing huge local fields enhancement at the surface of nanostructures, localized surface plasmon resonances (LSPRs) of the nanostructures have been investigated extensively [1,2,3]. LSPRs have a lot of applications in many fields such as surface enhanced Raman spectroscopy (SERS), optical switches, telecommunications, biosensing, photocatalytics, and optical traps [4,5,6,7,8,9,10]. The effective regulation of LSPRs is determined by the light polarization, structural parameters (size, shape), and surrounding environment of nanostructures [11,12,13,14]. Lately, the plasmonic Fano resonance that arises from the interaction between the dark plasmonic mode and the bright plasmonic mode is an interesting result of electro-magnetic coupling in nanostructures [15,16]. Because the bright plasmonic mode is similar to the continuum state and has finite dipole moments, it can be effectively excited by incident light. However, the dark plasmonic mode with zero dipole moments cannot be excited by incident light. Because of its high sensitivity characteristics, plasmonic Fano resonance has been widely studied and has been used for SERS, biosensing, nonlinear optics, and so on [17,18,19,20].

As a tunable nanostructure, a metallic nanoring-disk can effectively produce plasmonic Fano resonances [19,20,21,22,23,24,25,26]. Hao et al. found that symmetry-breaking in this nanostructure can lead to the interaction between the bright dipolar plasmonic mode and the dark quadrupolar plasmonic mode and form tunable Fano resonances [21,22]. Fu et al. reported that higher order Fano resonances were induced in a disk-ring nanosystem when the size of the nanodisk is reduced to a certain scale [23]. Zhang et al. proposed the formation mechanism of the narrow dark plasmonic mode, which indicated that such a structure can induce narrow, high contrast-ratio plasmonic Fano resonance [24]. Yi et al. reported that the Fano resonances were induced in concentric Ag nanoring–nanodisk without symmetry-breaking [25]. The metallic nanoring-disk nanosystems have been designed to induce tunable Fano resonances, which have the great prospect of applications in sensing detection [26,27,28]. Recently, it was found that the excitation and coupling of multiple plasmon modes could also result in multipolar Fano resonances responses [29,30,31]. However, effective and independent regulation of these multipolar Fano resonances is still a huge challenge, which will limit their application.

Inspired by the work of previous work [32,33,34], in this research, we designed Au ring-disk nanostructures with the disk outside of the ring to research the multipolar plasmonic Fano resonance. Compared to the reported research work, the multiple Fano resonance of our designed nanostructures is easier to be adjusted by regulating the structural parameters. The multipolar plasmonic Fano resonances, supported by different structural parameters, the Au ring-disk nanostructure is excited, which can be effectively tuned. Using different geometry parameters of Au ring-disk nanostructures, we can shift their Fano resonances with a certain channel, which have a lot of potential applications in plasmonic switches and chemical sensing.

## 2. Theoretical Methods

**Figure 1:**
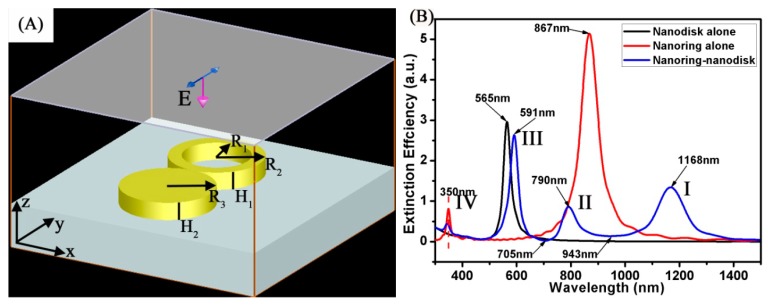
(**A**) Schematic illustration of the Au ring-disk nanostructures used in the simulations. (**B**) The spectrum calculated for the Au disk alone (black curve), Au ring alone (red curve), and Au ring-disk nanostructures (blue curve).

The multipolar plasmonic Fano resonances of the Au ring-disk plasmonic nanostructures were calculated by the finite-difference time-domain method [35]. A plane wave polarized across the y-direction in Figure 1 was illuminated from the SiO_2_ substrate, perpendicular to the Au ring-disk (as shown in Figure 1A). The boundary condition of the calculation perfectly matched the layer absorbing boundaries. The simulation volume was 140 × 140 × 140 nm^3^, and the cell size was 0.5 × 0.5 × 0.5 nm^3^. Here, the dielectric function of the Au ring-disk was taken from Johnson and Christy [36]. The refractive index of the surrounding medium was set to be 1.0. The dielectric function of the SiO_2_ was taken from Palik [37]. The near-field intensity enhancement pictures were drawn by dividing the electric field strength (E^2^ = Ex^2^ + Ey^2^ + Ez^2^) around the Au ring-disk. Figure 1A displays the schematic geometries of the Au ring-disk plasmonic nanostructures. As shown in Figure 1A, the structural parameters of the Au ring-disk plasmonic nanostructures were as follows: outer radius (R_2_), inner radius (R_1_), and thickness (H_1_)) and disk (radius (R_3_) and thickness (H_2_). In all our research work, we define the height (H_1_ = H_2_) as equal.

## 3. Results and Discussion

The calculated extinction spectrum of different models is shown in Figure 1B. In this case, the structural parameters of the Au ring were as follows: R_2_ was 30 nm, R_1_ was 20 nm, and H_1_ was 10 nm. The structural parameters of Au disk were as follows: R_3_ was 30 nm and H_2_ was 10 nm. The polarization direction of the plane wave was parallel to the y-axis. For a single disk, the extinction spectrum (the black curve) only had a single dipole resonance peak around 565 nm with a strong intensity [38]. For a single ring, the extinction spectrum (the red curve) shows the transverse dipole bonding mode around 867 nm, which came from the symmetric coupling between the internal and external surfaces of the Au ring dipolar modes [25,39]. For a ring-disk nanostructure, the extinction spectrum (the blue curve) has different plasmon resonances, indicating the electric dipole oscillation. The extinction spectrum of an Au ring-disk nanostructure shows four surface plasmon resonance (SPR) peaks. The definition of peaks is as follows: mode I around 1168 nm (transverse dipole bonding, the bright plasmonic mode), mode II around 790 nm (the dark plasmonic mode), mode III around 591 nm (the bright plasmonic mode), mode IV around 350 nm (the dark plasmonic mode). The extinction spectrum (the blue curve) has two apparent dips around 943 nm and 705 nm. The dips around 943 nm and 705 nm are spectrally overlapped by the dark plasmonic mode and the bright plasmonic mode. Generally speaking, Fano resonance generally produces an asymmetric resonance lineshape. The spectra that Zhang et al. reported has very asymmetric line shapes [39]. However, in our research work, the disk and the ring were in contact with each other. When the plane wave acts, the charge would transfer in both of the two, which would eventually make the spectra look not so asymmetric. The excellent dark plasmonic modes induced in the Au ring-disk nanostructure made them a good choice to interact with other bright plasmonic modes [40], where the multipolar Fano resonances can be easily tuned.

**Figure 2:**
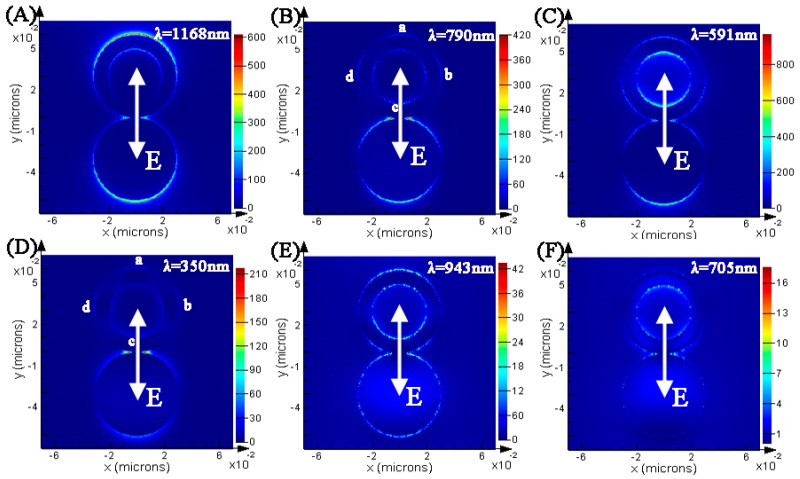
(**A**–**F**) Calculated electric field |E^2^|/|E_0_^2^| of the Au ring-disk nanostructures at different SPR peaks. H_1_ = H_2_ = 10 nm, R_1_ = 20 nm, R_2_ = 30 nm, and R_3_ = 30 nm. The color bars in the logarithmic scale for electric enhancement are shown (the units are (V/m)^2^, E_0_ = 1 V/m).

Figure 2 shows the field distributions of Au ring-disk nanostructure surfaces at different modes of resonance peaks, with four SPR peaks located around 1168, 790, 591 and 350 nm, and two dips around 943 and 705 nm. The position where the cross-sectional views of the electric field distribution cut at the center of the ring-disk. The field distributions in Figure 2A show the enhanced local field of the SPR peak around 1168 nm. The field distributions of the dipole are mainly along the y polarization. The field distributions are mainly concentrated around the outer surface of Au ring and around the disk. As shown in Figure 2B, the strong field distributions were mainly distributed around the disk and displayed a dipole plasmon mode. The weak field distributions on the Au ring exhibited four nodes, which meant the existence of a quadrupole plasmon mode; therefore, the 790 nm of the Au ring was a dark plasmonic mode. As shown in Figure 2C, the strong field distributions appeared around the disk and at the inner surface of the ring. As shown in Figure 2D, the strong field distributions were concentrated at the junction between the ring and the disk. Furthermore, the field distributions on the ring exhibited four nodes, which meant the existence of a quadrupole plasmon mode, therefore the peak around 350 nm was a dark plasmonic mode. As we know, the coupling of bright and dark plasmonic modes can produce Fano resonance. The Figure 2E,F display the field distributions around 943 nm and 705 nm. According to an analysis, SPR peaks around 1168, 790, 591, and 350 nm, and dips around 943 and 705 nm, supplied the possibility to inducing Fano resonances. The field distributions (see in Figure 2E) around 943 nm exhibited a chaotic distribution. The field distributions along and perpendicular to the incident polarization are disappeared, and the field distributions at the disk and ring presented a multipolar distribution. Therefore, the bright plasmonic mode around 1168 nm and dark plasmonic mode around 790 nm would excite the appearance of Fano resonance around 943 nm. The multipolar field distributions (Figure 2F) mainly appeared on the inner surface of the ring. As we know, the complex field distributions can be excited because of the coupling of bright and dark plasmonic modes [41].

As we know, the multipolar Fano resonances are excited when the bright and dark plasmonic modes interact and overlap in energy. In order to achieve effective spectral overlap between the bright and dark modes, we should carefully optimize the structural parameters of nanostructures. In the following, we confirm that the multipolar Fano resonances can be effectively modified by changing the geometric parameters of the Au ring-disk nanostructure, such as (1) the R_1_ of the Au ring, (2) the R_2_ of the Au ring, (3) the R_3_ of the Au disk, and (4) the H of the Au ring-disk nanostructure.

**Figure 3:**
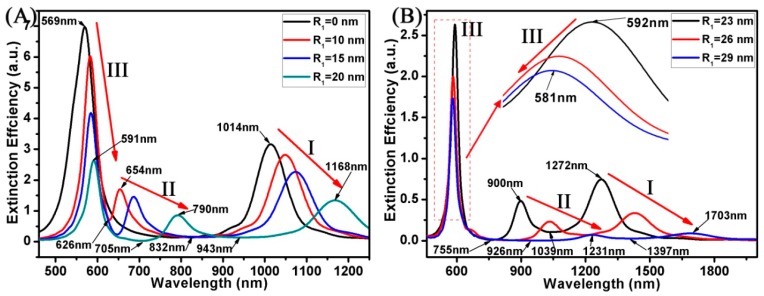
(**A**,**B**) Spectra calculated for the Au ring-disk nanostructures with different R_1_. The R_2_ of the ring was unchanged (R_2_ = 30 nm). The R_3_ of the disk was unchanged (R_3_ = 30 nm). The H of the ring-disk nanostructures was unchanged (H_1_ = H_2_ = H = 10 nm). The R_1_ of the ring increased from 0 to 29 nm.

Figure 3 displays the extinction spectrum of the Au ring-disk nanostructures with different R_1_s for the Au ring. In this case, the structural parameters of the Au ring were as follows: H_1_ was 10 nm, and R_2_ was 30 nm. The structural parameters of the Au disk were as follows: H_2_ was 10 nm, and R_3_ was 30 nm. When the R_1_ was 0 nm, the composite system consisted of two linked Au disks. As shown in Figure 3A, the curve has two SPR peaks around 569 nm and 1014 nm. However, when the R_1_ varied from 10 to 29 nm, these curves displayed three SPR peaks at mode I, mode II, and mode III. When the R_1_ varied from 0 to 29 nm, the SPR peaks of mode I were red shifted from 1014 to 1703 nm. The relative intensity of the SPR peaks reduced from 3.16 to 0.09. For the Au nanoring, the SPR peaks red shifted and the relative intensity of the SPR peaks increased with increasing thickness (t = R_2_ − R_1_) of the nanoring [42,43]. When other parameters of the Au ring-disk nanostructure were fixed, the t increased sharply while the R_1_ of the Au ring decreased. Free electrons redistributed on the Au ring-disk nanostructure, and the bonding strength increased. Therefore, the relative intensity of mode II decreased with increasing the R_1_ of the ring, which decreased from 1.7 to 0.07. When the R_1_ of the ring varied from 10 to 29 nm, a red shift of mode II resonances occured and the extinction efficiency decreased. The mode II resonances were red shifted from 654 nm (R_1_ = 10 nm) to 1231 nm (R_1_ = 29 nm). The extinction efficiency of mode II resonances decreased from 1.7 to 0.07. When the R_1_ of the ring varied from 0 to 23 nm, the mode III resonances were red shifted from 569 to 592 nm. However, when the R_1_ of the ring varied from 23 to 29 nm, the mode III resonances were blue shifted from 592 to 581 nm. Furthermore, the extinction efficiency of the mode III resonances decreased from 6.96 to 1.73. The corresponding extinction spectrum of the Au ring-disk nanostructures exhibited two modes of Fano resonances. With the R_1_ of the Au ring increasing from 10 to 29 nm, one mode of Fano resonances were red shifted from 626 to 926 nm, and another mode of Fano resonances were red shifted from 832 to 1397 nm. These wide spectra modulations from visible to near infrared wavelengths have important applications in sensing and detection.

**Figure 4:**
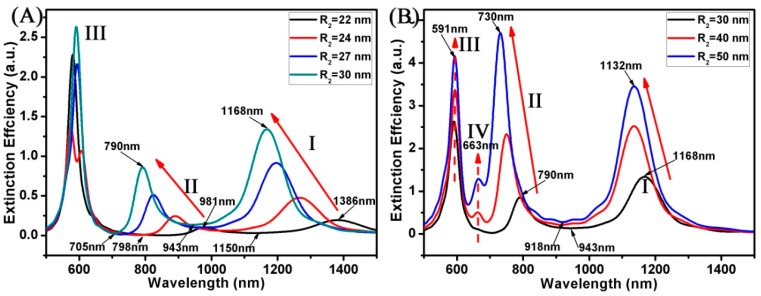
(**A**,**B**) Spectra calculated for the Au ring-disk nanostructures with different R_2_. The R_1_ of the ring was unchanged (R_1_ = 20 nm). The R_3_ of the disk was unchanged (R_3_ = 30 nm). The H of the ring-disk nanostructures was unchanged (H_1_ = H_2_ = H = 10 nm). The R_2_ of the ring increased from 22 to 50 nm.

Figure 4 displays the extinction spectrum of the Au ring-disk nanostructures with different R_2_. In this case, the structural parameters of the Au ring were as follows: H_1_ was 10 nm, and R_1_ was 20 nm. The structural parameters of the Au disk were as follows: H_2_ was 10 nm, and R_3_ was 30 nm. The R_2_ of the ring increased from 22 to 50 nm. When the R_2_ of the Au ring varied from 22 to 50 nm, as shown in Figure 4, these resonance peaks (mode I and mode II) were blue shifted, and the extinction efficiency increased. For larger R_2_, the blue shift of mode I and mode II appeared with the increase of the t of the ring (t = R_2_ − R_1_). When the R_2_ of the ring was 22 nm, the resonance peak (mode I) appeared around 1386 nm, which was a blue shift to 1132 nm once the R_2_ increased to 50 nm. When the R_2_ of the ring was 22 nm, the resonance peak (mode II) appeared around 981 nm, which was blue shifted to 730 nm once the R_2_ increased to 50 nm. When R_2_ was less than or greater than 30 nm, the variation trend of mode I and mode II was somewhat different. When the R_2_ was less than 30 nm, the small change of R_2_ would cause the obvious change of the SPR peaks’ (mode I and mode II) positions. For an R_2_ greater than 30 nm, the trend would slow down. These results made the variation trend of Fano resonances also have a very big difference. When the R_2_ was less than 30 nm, Fano resonances were blue shifted from 1150 to 943 nm. However, when the R_2_ was greater than 30 nm, Fano resonances were blue shifted from 943 to 918 nm. This indicates that when the R_2_ was larger than the R_3_ of the Au disk, the Fano resonances were not able to be adjusted. When the R_2_ varied from 30 to 50 nm (R_2_ > R_3_), some new multipolar SPR peaks around 663 nm (mode IV) appeared in these curves.

**Figure 5:**
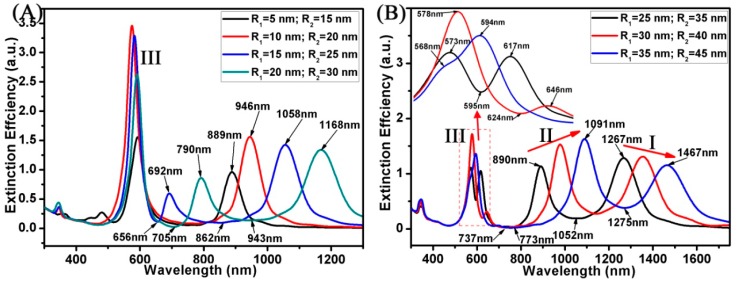
(**A**,**B**) Spectra calculated for the Au ring-disk nanostructures with different R_1_ and R_2_. The R_3_ of the disk was unchanged (R_3_ = 30 nm). The H of the ring-disk nanostructures was unchanged (H_1_ = H_2_ = H = 10 nm). The t of ring was unchanged (t = R_2_ − R_1_ = 10 nm).

Figure 5 shows the extinction spectrum of the Au ring-disk nanostructures with different R_1_ and R_2_. The thickness (t = R_2_ − R_1_) of the Au ring was fixed at 10 nm. The R_3_ of the Au disk was fixed at 10 nm, and the H of the Au ring-disk nanostructures (H_1_ = H_2_ = H) was fixed at 10 nm. When the radius of the Au ring was relatively small (R_2_ < 20 nm), as shown in Figure 5A, these curves only had two resonance peaks. This result is similar to the result shown in Figure 3A with two Au nanodisks. This indicates that the Au ring had a smaller radius, and the effect was similar to that of the two Au nanodisks. When the radius of the Au ring was relatively big (R_2_ > 20 nm), these curves show multipolar SPR peaks. Here, when the R_2_ of Au ring increased, the SPR peaks of mode I were red shifted from 1058 to 1467 nm, and the SPR peaks of mode II were red shifted from 692 to 1091 nm. With the increase of R_1_ and R_2_, Fano resonances in the short wavelength region were red shifted from 656 to 773 nm. The Fano resonances in the long wavelength region were red shifted from 862 to 1275 nm with the increase of R_1_ and R_2_. The high tunability of the Au ring meant we could change its radius to tune the Fano resonances.

**Figure 6:**
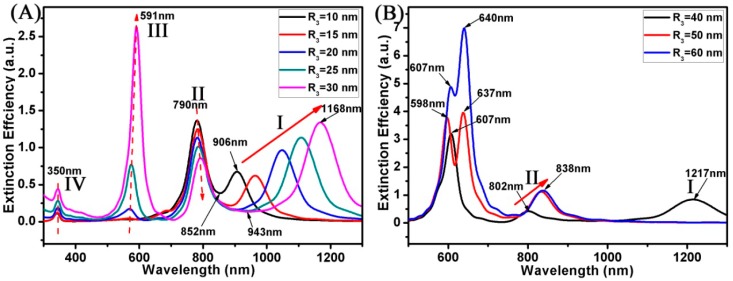
(**A**,**B**) Spectra calculated for the Au ring-disk nanostructures with different R_3_. The R_1_ and R_2_ of the ring was unchanged (R_1_ = 20 nm; R_2_ = 30 nm). The H of the ring-disk nanostructures was unchanged (H_1_ = H_2_ = H = 10 nm). The R_3_ of the disk increased from 10 to 60 nm.

As we know, the optical properties of the Au disk are mainly governed by the dipolar mode [44,45]. Here, we can also predict that the R_3_ of disk will affect the optical properties of Au ring-disk nanostructures. Figure 6 shows the extinction spectrum of the Au ring-disk nanostructures with different R_3_s of the Au disk. In this case, the structural parameters of the Au ring were as follows: R_1_ was 20 nm and R_2_ was 30 nm. The H of the ring-disk nanostructures was fixed at 10 nm (H_1_ = H_2_ = H = 10 nm). As shown in Figure 6, the variation trend of the SPR peaks shows two changes. As shown in Figure 6A, when the radius of the Au disk was relatively small (R_3_ ≤ 30 nm), these curves had four modes of resonance peaks. In the SPR peaks of these four modes, only the peak position of mode I changed with the change of the R_3_. When the R_3_ varied from 10 to 30 nm, the SPR peaks of mode I were red shifted from 906 to 1168 nm. The relative intensity of mode I increased from 0.67 to 1.34. For resonance peaks of the II, III, and IV modes, the position of these SPR peaks was not adjusted with the change of the R_3_, which were kept around 790 nm, 591 nm, and 350 nm, respectively. For resonance peaks of the III mode, the relative intensity increased from 0 to 2.61 with the increase of R_3_. This shows that the coupling effect between the disk and the ring increased with the increase of R_3_. As shown in Figure 6A, with the increase of R_3_, these Fano resonances were red shifted from 852 to 943 nm. When the radius of the Au disk was relatively large (R_3_ > 30 nm), as shown in Figure 6B, these curves show different extinction characteristics compared to Figure 6A. When R_3_ varied from 40 to 60 nm, the SPR peaks of mode I disappeared and the SPR peaks of mode II were red shifted from 802 to 838 nm. When R_3_ was further enlarged, such as R_3_ = 50 nm, the SPR peaks at mode III gave multiple peaks.

**Figure 7:**
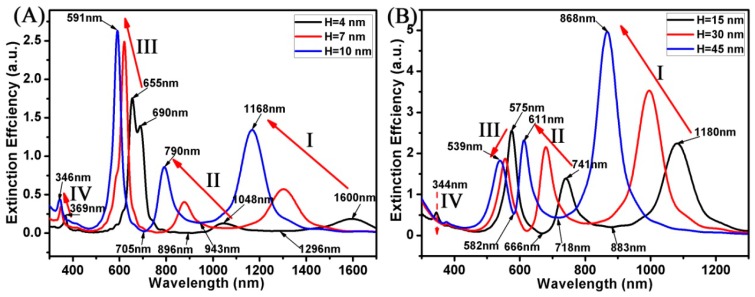
(**A**,**B**) Spectra calculated for the Au ring-disk nanostructures with different H. The R_1_ and the R_2_ of the Au ring was unchanged (R_1_ = 20 nm; R_2_ = 30 nm). The R_3_ of the disk was 30 nm. The H increased from 4 to 45 nm.

As we know, the optical properties of Au nanostructures have a significant relationship with their thickness [46,47]. In this case, the structural parameters of the Au ring were as follows: R_1_ was 20 nm and R_3_ was 30 nm. The structural parameters of the Au disk were as follows: R_3_ was 30 nm. The H of the Au ring-disk nanostructures varied from 4 to 45 nm. Figure 7 shows the extinction spectrum of the Au ring-disk nanostructures with different H. Four modes of resonance peaks (I, II, III, and IV modes) could be excited in these extinction spectra. When the H varied from 4 to 45 nm, the SPR peaks of I mode was blue shift from 1600 to 868 nm, the SPR peaks of II mode were blue shifted from 1048 to 611 nm. This indicates that the thin Au ring-disk nanostructures with other parameters fixed had a larger optical extinction cross section. When the H became higher, the intensity of I mode increased from 0.18 to 4.96 and the intensity of II mode increased from 0.13 to 2.31. For calculations whose purpose was to study higher multipolar coupling, the Au ring-disk nanostructure with a higher H had the advantage due to its stronger intensity. The result was also the same as the nanoring where different plasmon resonances resulted in the phase retardation because the H of nanoring was increased [48]. For the resonance peaks of III mode, when the H varied from 4 to 45 nm, the SPR peaks were blue shifted from 690 to 539 nm. However, the intensity shows a Gaussian distribution, the Au ring-disk nanostructures (H = 10 nm) had the better response to the incident light, and the intensity was 2.63. Here, when the height was smaller (such as H = 4 nm), the extinction spectrum (III mode) shows the multipolar SPR peaks (655 nm and 690 nm). For the Fano resonances, within the range of different H, the changes were different. When the H was smaller than 10 nm, one mode of the Fano resonances were blue shifted from 1296 to 943 nm, and another mode of Fano resonances were blue shifted from 896 to 705 nm. However, when the H of the Au ring-disk nanostructures was bigger than 10 nm, one mode of Fano resonances was blue shifted from 883 to 718 nm, and another mode of Fano resonances was blue shifted from 666 to 582 nm. This shows that these Fano resonances were easier to adjust when the H of the Au ring-disk nanostructures was relatively thin.

## 4. Conclusions

In summary, we have theoretically demonstrated the tunable multipolar Fano resonances and near field properties in Au ring-disk nanostructures. These multipolar Fano resonances were oriented from the interaction between the dark plasmonic mode and bright plasmonic mode. The parametric studies revealed that these structural parameters, such as the radius (R_1_ and R_2_) of the Au ring, the radius (R_3_) of the Au disk, and the thickness (H) of the Au ring-disk nanostructures, played a very important role in controlling the multipolar Fano resonances. With the R_1_ of Au ring increasing from 10 to 29 nm, one mode of Fano resonances was red shifted from 626 to 926 nm, and another mode of Fano resonances was red shifted from 832 to 1397 nm. When the R_2_ was less than 30 nm, Fano resonances were blue shifted from 1150 to 943 nm. However, when the R_2_ was greater than 30 nm, Fano resonances were blue shifted from 943 to 918 nm. With the increase of R_1_ and R_2_, Fano resonances in the short wavelength region were red shifted from 656 to 773 nm. The Fano resonances in the long wavelength region were red shifted from 862 to 1275 nm with the increase of R_1_ and R_2_. When the R_3_ varied from 10 to 30 nm, these Fano resonances were red shifted from 852 to 943 nm. When the H was smaller than 10 nm, one mode of the Fano resonances was blue shifted from 1296 to 943 nm, and another mode of Fano resonances was blue shift from 896 to 705 nm. However, when the H of the Au ring-disk nanostructures was bigger than 10 nm, one mode of Fano resonances was blue shifted from 883 to 718 nm, and another mode of Fano resonances was blue shifted from 666 to 582 nm. By regulating structural parameters, these tunable multipolar Fano resonances have wide regulation from visible to near infrared wavelengths. This new nanocomposite structure is expected to have potential applications in plasmonic switching and chemical sensing.
